# Aflatoxin B_1_ Exposure in Sheep: Insights into Hepatotoxicity Based on Oxidative Stress, Inflammatory Injury, Apoptosis, and Gut Microbiota Analysis

**DOI:** 10.3390/toxins14120840

**Published:** 2022-12-01

**Authors:** Yuzhen Sui, Ying Lu, Shoujun Zuo, Haidong Wang, Xiaokun Bian, Guizhen Chen, Shucheng Huang, Hongyu Dai, Fang Liu, Haiju Dong

**Affiliations:** International Joint Research Center of National Animal Immunology, College of Veterinary Medicine, Henan Agricultural University, Zhengzhou 450046, China

**Keywords:** Aflatoxin B_1_, liver function, oxidative stress, inflammatory, apoptosis, gut microbiota

## Abstract

The widespread fungal toxin Aflatoxin B_1_ (AFB_1_) is an inevitable pollutant affecting the health of humans, poultry, and livestock. Although studies indicate that AFB_1_ is hepatotoxic, there are few studies on AFB_1_-induced hepatotoxicity in sheep. Thus, this study examined how AFB_1_ affected sheep liver function 24 h after the animals received 1 mg/kg bw of AFB_1_ orally (dissolved in 20 mL, 4% *v*/*v* ethanol). The acute AFB_1_ poisoning caused histopathological injuries to the liver and increased total bilirubin (TBIL) and alkaline phosphatase (AKP) levels. AFB_1_ also markedly elevated the levels of the pro-inflammatory cytokines TNF-α and IL-6 while considerably reducing the expression of antioxidation-related genes (*SOD-1* and *SOD-2*) and the anti-inflammatory gene *IL-10* in the liver. Additionally, it caused apoptosis by dramatically altering the expression of genes associated with apoptosis including *Bax*, *Caspase-3*, and *Bcl-2*/*Bax*. Notably, AFB_1_ exposure altered the gut microbiota composition, mainly manifested by *BF311* spp. and *Alistipes* spp. abundance, which are associated with liver injury. In conclusion, AFB_1_ can cause liver injury and liver dysfunction in sheep via oxidative stress, inflammation, apoptosis, and gut-microbiota disturbance.

## 1. Introduction

Mycotoxins are widespread in all processes of agricultural production and can seriously endanger food and feed safety, threatening human and animal health [1]. Over 4.5 billion people are at high risk of exposure to these food contaminants, and it causes annual economic losses of hundreds of billions of dollars. According to the Food and Agriculture Organization of the United Nations (FAO), about 25% of the world’s crops are polluted by mycotoxins in varying degrees [2,3]. Aflatoxin B_1_ (AFB_1_), the most toxic mycotoxin, is hepatotoxic, teratogenic, and mutagenic, and it is classified as a class I carcinogen by the World Health Organization [4,5]. The sensitivity of different livestock species to AFB_1_ varies [6,7]. Chickens are highly sensitive to AFB_1_, and poisoning is caused mostly by long-term consumption of AFB_1_-contaminated feed [5,7]. Although sheep are typically thought to have significant tolerance to AFB_1_ due to their status as ruminants, poisoning can, nonetheless, occur from prolonged or excessive consumption of an AFB_1_-containing diet [8].

As a hepatotoxic chemical substance, AFB1 is activated into AFB1 epoxide under the action of the cytochrome P450 (CYP450) enzyme, inducing liver cancer and hepatotoxicity [9]. AFB1 toxicity can lead to oxidative damage, apoptosis, and inflammation, resulting in liver congestion, pale coloration, enlargement, and necrosis, inducing chronic and acute hepatocellular injury [7,10,11,12]. Livestock and poultry can accumulate a certain level of AFB_1_ through dietary exposure, leading to chronic and acute liver diseases in humans via biological accumulation in the food chain [13].

The liver is an important detoxification center as well as a target organ for the metabolic transformation of AFB_1_ in the body, and it is crucial for the body’s defense against xenobiotics [14]. Past studies on the hepatotoxic effects of AFB_1_ exposure in sheep have not explored the association between changes in liver function indicators and oxidative stress, inflammation, apoptosis, or gut microbiota. Therefore, we sought to assess the liver function, oxidation, inflammation, and apoptosis indices and composition of the fecal microbial community to offer theoretical support and experimental data on liver damage caused by AFB_1_ exposure in sheep.

## 2. Results

### 2.1. Effects of AFB_1_ Exposure on Clinical Symptoms, Body Temperature, Respiration, Heart Rate, and Conjunctival Color

Sheep in the AFB_1_ group showed poor mental status, foaming at the mouth, and reduced food intake after gavage compared to the control group. Before AFB_1_ exposure, there were no significant differences between the control and AFB1 group body temperatures, respiration, or heart rates, as indicated in Table S2 (*p* = 0.280, 0.280, and 0.146, respectively). Nevertheless, 24 h after AFB_1_ exposure, the respiration and heart rate of the AFB1 group were lower than those of the control group (*p* = 0.071 and 0.084, respectively), while the body temperature was higher (*p* = 0.519). The AFB_1_ group had lower changes (slope) in respiration and heart rate than the control group (*p* = 0.488 and 0.022, respectively). Furthermore, the temperature change in the AFB_1_ group was greater than in the control group (*p* = 0.043).

As can be observed in Figure S1A–C, the conjunctival color L of the AFB1 group dropped considerably (*p* = 0.005) after acute exposure compared with the control group, although redness (a value) and yellowness (b value) did not differ significantly (*p* = 0.442 and 0.262, respectively).

### 2.2. Effects of AFB_1_ Exposure on Serum Biochemical Indices 

As shown in Table S3, serum calcium (Ca) levels in the AFB_1_ group were considerably lower (*p* = 0.006) than those in the control group, while serum total protein (TP), albumin (ALB), globulin (GLO), white sphere ratio (A/G), amylase (AMY), phosphorus (P), and glucose (GLU) levels showed insignificant decreasing trends (*p* > 0.05). Furthermore, serum total bilirubin (TBIL) levels were considerably higher (*p* = 0.002), and serum alanine aminotransferase (ALT), aspartate aminotransferase (AST), creatinine (CRE), urea nitrogen (BUN), BUN/CRE, and creatine kinase (CK) levels increased in the AFB_1_ group compared with the control group (*p* = 0.297, 0.190, 0.306, 0.599, 0.938, and 0.41, respectively). The increased serum indices of AST, ALT, and TBIL suggests that AFB_1_ exposure could result in liver dysfunction (Table S3).

### 2.3. Effects of AFB_1_ Exposure on Liver Tissue Structure and Liver Tissue Function

**Figure 1:**
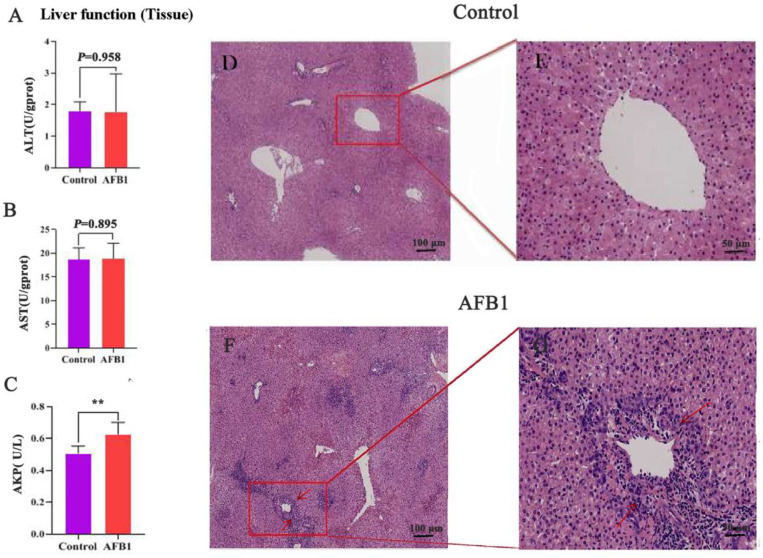
Effects of AFB_1_ exposure on liver function and morphological structure of liver tissue. (**A**–**C**) Liver function indexes of liver tissue (ALT, AST, and AKP). (**D**,**E**) H&E staining reveals normal liver tissues. (**F**,**G**) Liver morphology after AFB_1_ exposure. Note the presence of ruptured cells and inflammatory cell infiltration (arrow). 100 μm (**D**,**F**), 50 μm (**E**,**G**). ** *p* < 0.01.

As shown in Figure 1A–C, the ALT, AST, and AKP levels in the AFB_1_ group increased when compared with those of the control group. Among them, the AKP level was considerably higher than that in the control group (*p* = 0.007).

Figure 1D–G depicts liver tissue stained with H&E. The liver lobules of the control group had normal shape; the cords were arranged in an orderly, radial pattern; and the liver cells were complete and uniform in size and cytoplasm. The liver tissue of the AFB_1_ group, in contrast, exhibited pathological changes, including abnormal hepatic lobule structure, disorganized hepatic cord arrangement, swollen and unevenly sized hepatocytes, severe infiltration of inflammatory cells, vacuolar degeneration, and necrosis in some hepatocytes.

### 2.4. Effects of AFB_1_ Exposure on Liver Oxidative Damage

**Figure 2:**
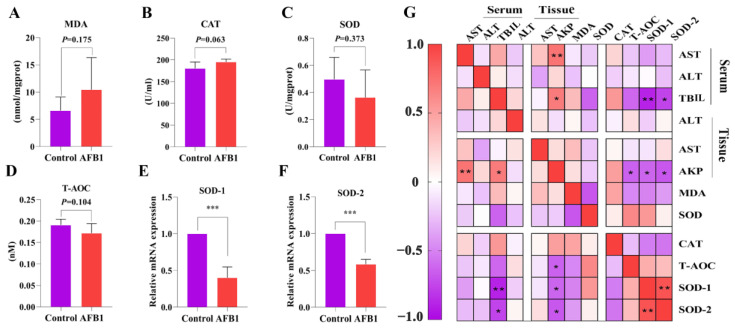
Effects of AFB_1_ exposure on liver oxidative damage in sheep. (**A**–**D**) Liver tissue MDA, CAT, SOD, and T-AOC levels. (**E**,**F**) Relative expressions of the genes *SOD-1*, and *SOD-2* related to oxidative stress in the liver. (**G**) Pearson correlation analysis of oxidative stress indexes and parameters related to liver function. * *p* < 0.05, ** *p* < 0.01, *** *p* < 0.001.

A decrease in liver function is related to oxidative stress [15]. Measurements were taken of the concentrations of MDA and T-AOC and the activities of antioxidant enzymes (CAT, SOD). There was no significant difference in MDA, SOD, CAT, and T-AOC levels between the two groups (*p* > 0.05; Figure 2A–D). Notably, MDA concentration and SOD activity showed an increasing and decreasing trend compared with the control group, respectively. Protein and mRNA levels typically show reasonable correlation [16], so we estimated the expression of *SOD-1* and *SOD-2* genes in liver tissues. *SOD-1* and *SOD-2* levels in the AFB_1_ group were considerably lower than those in the control group (*p* < 0.001; Figure 2E,F).

The relationship between antioxidant indexes and liver function-related indexes was investigated using Pearson correlation analysis (Figure 2G). The levels of AKP (tissue) were negatively correlated with those of T-AOC, SOD-1, and SOD-2 (r = −0.639, −0.175, −0.411, and *p* = 0.025, 0.03, and 0.002, respectively). SOD-1 (r = −0.893, *p* = 0.001) and SOD-2 (r = −0.760, *p* = 0.029) levels were also negatively correlated with TBIL (serum).

### 2.5. Effects of AFB_1_ Exposure on the Expression of Inflammation-Related Factors in the Liver

**Figure 3:**
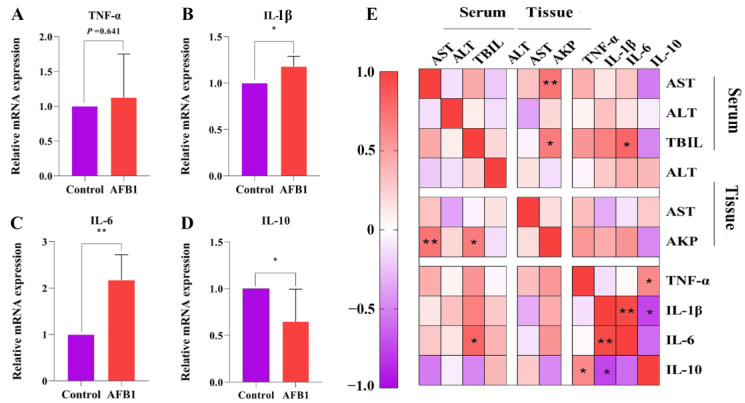
Effects of AFB_1_ exposure on inflammation-related indexes in sheep. (**A**–**D**) TNF-α, IL-1β, IL-6, and IL-10 mRNA levels detected in liver. (**E**) Heat map showing the correlation between indicators of liver function and levels of inflammatory gene expression. * *p* < 0.05, ** *p* < 0.01.

To further investigate the effects of AFB_1_ on liver inflammation, we examined the expression of the inflammatory cytokines TNF-α, IL-6, and IL-1β and anti-inflammatory cytokine IL-10 in the liver tissues of sheep in each group (Figure 3A–D). The results indicate that the AFB_1_ group had significantly higher levels of IL-1β and IL-6 expression than those of the control group (*p* = 0.021 and 0.003, respectively). Furthermore, IL-10 expression was considerably lower in the AFB_1_ group (*p* = 0.034), although TNF-α expression did not change significantly (*p* = 0.641). Pearson correlation analysis revealed that *IL-6* gene expression was positively correlated to serum TBIL levels (r = 0.798 and *p* = 0.01; Figure 3E).

### 2.6. Effects of AFB_1_ Exposure on Liver Cell Apoptosis and Apoptosis-Related Gene Expression

**Figure 4:**
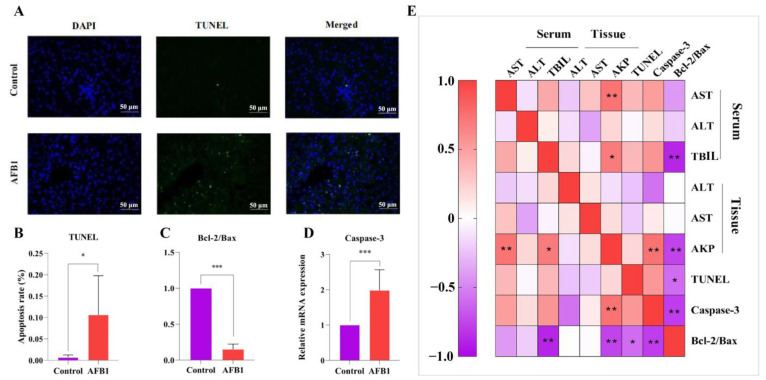
Effects of AFB_1_ exposure on hepatocyte apoptosis indexes in sheep. (**A**) TUNEL staining of sheep liver tissue, with green fluorescence indicating TUNEL positive cells and DAPI staining for nucleus (400×). (**B**) TUNEL-positive cell percentage. (**C**) Bcl-2 to Bax ratio. (**D**) Caspase 3 mRNA level detected in liver using RT-qPCR. (**E**) Heat map showing the correlation between indicators of liver function and hepatocyte apoptosis indexes. * *p* < 0.05, ** *p* < 0.01, *** *p* < 0.001.

TUNEL staining in the liver of sheep in each group is shown in Figure 4A,B. The AFB_1_ group had a considerably higher positive rate of TUNEL than the control group. We also detected the mRNA expression of Caspase-3, Bax, and Bcl-2 in the hepatocytes. As seen in Figure 4C,D, the Bcl-2/Bax ratio was reduced dramatically (*p* < 0.001) in the AFB_1_ group compared with the control group, whereas *Caspase-3* gene expression increased significantly (*p* = 0.002).

According to the Pearson correlation analysis, AKP (tissue) was negatively correlated with the Bcl-2/Bax ratio (r = −0.762 and *p* = 0.004) and positively correlated with the *Caspase-3* gene (r = 0.730 and *p* = 0.07; Figure 4E). The Bcl-2/Bax ratio and serum TBIL level had a negative correlation (r = −0.883 and *p* = 0.002; Figure 4E).

### 2.7. Changes in Gut Microbiota Induced by AFB_1_ Exposure

**Figure 5:**
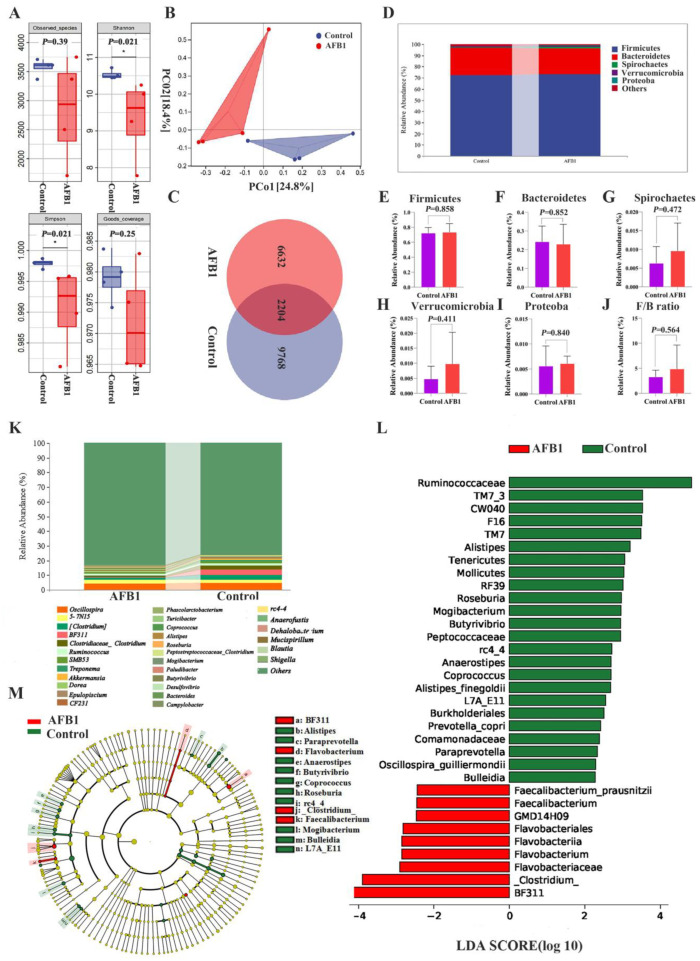
Effects of AFB_1_ exposure on gut microbiota structure. (**A**) Alpha diversity indexes (observed species, Shannon, Simpson, and Good’s coverage) of intestinal microbial. (**B**) Principal Coordination Analysis (PCoA) of the intestinal microbiome in the AFB_1_ and control groups (*n* = 4). (**C**) ASVs Venn diagram. (**D**) The top five phylum-level abundances of gut microbiota. (**E**–**J**) Difference in the abundances of gut microbiota (*Firmicutes*, *Bacteroidetes*, *Spirochaetes*, *Verrucomicrobia*, *Proteobacteria*, and F/B ratio, respectively) at the phylum level. (**K**) Gut microbiota relative abundance at the genus level (Top 30). (**L**,**M**) LDA score and cladogram of LDA effect size (LEfSe). * *p* < 0.05.

An increasing number of studies show that changes in gut microbiota composition and function are crucial for liver health [17]. To study how the composition of gut microbiota changed after AFB_1_ exposure, 16S rRNA gene sequencing was performed. The AFB1 and control groups had coverage rates of 98.39% and 96.46%, respectively, indicating that the majority of the gut microbiota diversity was detected (Figure 5A). In comparison to the control group, the observed species, Shannon, Simpson, and Good’s coverage indexes in the AFB1 group were reduced (*p* = 0.39, 0.021, 0.021, and 0.25, respectively; Figure 5A). The gut microbiota composition showed a trend of relative separation (*p* = 0.06) for Beta diversity between the control and AFB_1_ groups (PCo1 contribution of 27.80%, PCo2 contribution of 22.80%; Figure 5B). A total of 2204 ASVs were found in both groups, with 9768 and 6632 ASVs found in the control and AFB_1_ groups, respectively (Figure 5C). *Firmicutes*, *Bacteroidetes*, *Spirochaetes*, *Verrucomicrobia*, and *Proteobacteria* were the top five abundant phyla in both groups, as shown in Figure 5D. *Firmicutes*, *Spirochaetes*, *Verrucomicrobia*, and *Proteobacteria* were more abundant in the AFB_1_ group (73.49%, 0.96%, 0.99%, and 0.61%, respectively) than in the control group (72.23%, 0.56%, 0.49%, and 0.56%, respectively), while *Bacteroidetes* were more abundant in the control group (24.32%) than in the AFB_1_ group (23.01%; Figure 5D–I). The *Firmicutes*/*Bacteroides* ratio (F/B ratio) was higher in the AFB_1_ group than in the control group, but the difference was not significant (*p* = 0.564; Figure 5J).

The top 30 genera were also identified. Among them, *oscillospira* spp., *5-7N15* spp., and *(Clostridium)* spp. were the most predominant in the control group, and *oscillospira* spp., *5-7N15* spp., and *BF311* spp. accounted for the predominance in the AFB_1_ group (Figure 5K). LEfSe analysis was utilized to further detect the differential bacterial taxa between the two groups, and 33 discriminative features were identified (genus level; LDA score > 2, *p* < 0.05; Figure 5L). Among them, *BF311* spp., *Flavobacterium* spp., and *(Clostridium)* spp. were concentrated in the AFB_1_ group, while *Ruminococcaceae* spp., *Alistipes* spp., and *Roseburia* spp. were concentrated in the control group (Figure 5L,M).

**Figure 6:**
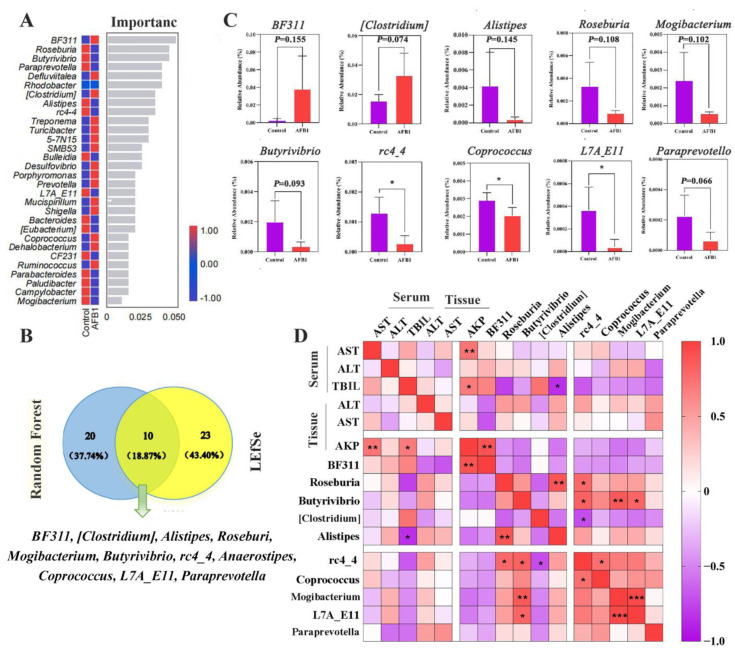
Analysis of differential gut microbiota. (**A**) Gut microbiota random forest analysis at the genera level (Top 30). (**B**) Venn diagrams for the LEfSe and the random forest. (**C**) The analysis of relative abundances of the ten genera between groups. (**D**) Heat map showing the correlation between indicators of liver function and gut microbiota. * *p* < 0.05, ** *p* < 0.01, *** *p* < 0.001.

The top 30 most important genera were screened using random forest analysis, and the most important species at the genus level were *BF311* spp., *Roseburia* spp., and *Butyrivibrio* spp. (Figure 6A). Thirty-three characteristic species were identified using a Venn diagram analysis of the LEfSe results (LDA score > 2; *p* < 0.05), and 10 genera were screened out from the top 30 genera based on significance scores (Figure 6B).

Furthermore, we noted that the abundances of *BF311* spp. and (*Clostridium*) spp. were higher in the AFB_1_ group, whereas those of *Alistipes* spp., *Roseburia* spp., *Mogibacterium* spp., *Butyrivibrio* spp., *rc4-4* spp., *Coprococcus* spp., *L7A_E11* spp., and *Paraprevotella* spp. were lower in the AFB_1_ group than in the control group (*p* = 0.155, 0.074, 0.145, 0.108, 0.102, 0.093, 0.015, 0.037, 0.022, and 0.066 respectively; Figure 6C). Pearson correlation analysis revealed that the TBIL (serum) level was significantly negatively correlated with *Alistipes* spp. (r = 0.84, *p* = 0.036; Figure 6D), whereas AKP (tissue) levels were positively correlated with *BF311* spp. (r = 0.897, *p* = 0.002; Figure 6D).

## 3. Discussion

Body temperature, breathing rate, and heart rate are important indicators of physical health [18]. In this study, the body temperatures of sheep increased and respiration and heart rates decreased after intake of AFB_1_. AFB_1_ exposure-induced gastrointestinal and neurological symptoms were also observed in this study. These results suggest that AFB_1_ exposure affects the health of sheep.

Jaundice is an important sign of liver dysfunction [19]. As AFB_1_ targets the liver, exposure to AFB_1_ may cause jaundice and change the color of the conjunctiva. Therefore, we measured conjunctival color and noted that AFB_1_ exposure significantly reduced the L values. However, no significant difference in b and a values was detected between the two groups, which could be attributed to the short-term exposure of the animals to AFB_1_ in this study.

The activities of serum enzymes such as ALT and AST, as well as TP, ALB, TBIL, and GLO concentration, have been defined as critical indicators of liver injury and function [20,21,22]. In this investigation, AFB_1_ exposure significantly increased the serum TBIL content of sheep, while the levels of serum TP, ALB, GLO, and A/G tended to decrease, and AST and ALT contents tended to increase. These phenomena suggest that exposure to AFB_1_ may harm the liver and impair liver function in sheep [23]. BUN and CRE are the main biochemical indexes of renal function, which increase with the aggravation of renal injury [24]. In this study, BUN and CRE levels tended to increase slightly, which may have been caused by the short acting time of AFB_1_ in this study. Ca^2+^ is an important signaling molecule involved in numerous cellular processes, and its content decreases with the aggravation of liver and kidney diseases [25]. AFB_1_ exposure significantly decreased the serum Ca content in sheep, indicating the possibility of liver damage. We next assessed liver tissue to explore whether AFB_1_ exposure would negatively affect sheep liver tissue.

AFB_1_ exposure resulted in hepatocyte degeneration and considerable inflammatory cell infiltration, suggesting liver tissue damage and corroborating the results of Tsiouris et al. (2021) [26]. In general, elevated ALT, AST, and AKP levels indicate liver dysfunction, which is critical for the differential diagnosis of liver diseases [27]. In the present study, AFB_1_ exposure increased ALT, AST, and AKP levels in the liver tissue of sheep compared with those of the control group, reflecting the negative impact of AFB_1_ exposure on liver function.

Numerous studies have demonstrated that AFB_1_ increases lipid peroxidation and induces the formation of high levels of reactive oxygen species and free radicals, damaging body organs through oxidative stress [28,29]. Since MDA is a byproduct of lipid peroxidation, the level of lipid peroxidation can be inferred from the MDA content in living things [30,31]. In this study, the MDA content increased slightly, indicating liver tissue injury. Antioxidant markers, including SOD, CAT, and T-AOC, are known to play key roles in attenuating oxidative stress by scavenging reactive oxygen species [32]. Although SOD and T-AOC levels were both reduced in the liver of sheep exposed to AFB_1_ compared to those of the control group, the difference was not significant. The activity of SOD, a particular antioxidant enzyme that neutralizes superoxide anions in ROS free radicals, indirectly reflects the capacity to neutralize oxygen free radicals and is crucial in liver injury [33]. Further examination of gene expression revealed that *SOD-1* and *SOD-2* levels were significantly decreased. However, an apparent increase in the CAT content was observed in the present study, which may have resulted from the defense mechanism of the body against AFB_1_ toxicity [8,34]. In this context, Cao et al. (2021) discovered that AFB_1_ intoxication significantly increases CAT activity in sheep exposed with AFB_1_ [8]. In addition, the correlation between liver function and antioxidant enzyme activity revealed that AFB_1_ exposure causes a decrease in liver function related to oxidative damage.

The inflammatory response is an important mechanism in AFB_1_ toxicity [35]. Oxidative stress can increase the production of different types of ROS in the body, and ROS can activate the nuclear factor-kappa B (NF-κB) pathway, leading to the production of inflammatory cytokines [36]. TNF-α is the most prominent pro-inflammatory cytokine involved in the activation of NF-κB, which induces the expression of IL-1β, IL-6, and other downstream inflammatory mediators [37]. AFB_1_ significantly increased the expression of the inflammatory factors IL-1β and IL-6, according to our findings. Furthermore, the anti-inflammatory *IL-10* gene, which is linked to liver function, was drastically downregulated. The changes in the inflammatory factor expression levels in the liver tissue imply that exposure to AFB_1_ causes inflammatory injury to the liver.

It has been reported that AFB_1_ destroys the integrity of the cell membrane by stimulating phospholipids and inducing ROS formation [38]. When excessive ROS is produced and the scavenging capacity of the body decreases, it can lead to protein, DNA, and mitochondrial damage, thus inducing apoptosis [39]. Excessive apoptosis can lead to organ damage, which is considered one of the mechanisms of AFB_1_-induced toxicity [40]. Our findings showed that when compared with the control group, the AFB_1_ group had a significantly higher rate of hepatocyte apoptosis detected using the TUNEL method. These results are similar with the findings of a prior study by Xu et al. (2021) [41]. The release of mitochondrial cytochrome c is affected by changes in the ratio of Bcl-2 to Bax expression in cells, and a decrease in this ratio results in apoptosis, according to studies conducted on mammals [42,43]. At the same time, caspase-3 is a common effector of apoptosis [44]. In this study, we discovered that after AFB_1_ exposure, Bcl-2/Bax gene expression decreased while caspase-3 gene expression increased, indicating that AFB_1_ exposure can promote liver cell apoptosis. In addition, the correlation between liver function and apoptosis showed that the decline in liver function caused by AFB_1_ exposure is related to apoptosis.

Like most mycotoxins, AFB_1_ not only directly damages body organs but also interferes with the normal activities of animal intestinal flora via enterohepatic circulation [7,8]. For example, long-term feeding of AFB_1_ can significantly reduce most intestinal microbiota in mice [45], and microbiota decline was observed in the acute AFB_1_ poisoning experiment in this study. Moreover, intestinal flora can combine, transform, degrade, and transfer mycotoxins; promote the healthy growth of livestock and poultry; and participate in material metabolism [46,47,48]. Through amplicon sequencing, we discovered that *Firmicutes* and *Bacteroidetes* were the two largest phyla that made up the sheep intestinal flora, which is consistent with earlier research on mammalian intestinal flora [49]. *Firmicutes*/*Bacteroidetes* ratios are typically correlated with inflammatory marker levels and pathological conditions of intestinal metabolic homeostasis [49]. AFB_1_ exposure significantly raised the F/B ratio in this study, implying that AFB_1_ exposure may disrupt gut metabolic homeostasis and alter inflammatory metabolite levels. At the same time, by screening at the genus level, we identified two genera that deserve attention: *BF311* spp. and *Alistipes* spp. Although there are few studies on the function of *BF311* spp., some suggest that *BF311* spp. may play a crucial role in the rumen ecosystem and even in rumen synchronization [50]. As a relatively newly identified bacterial genus, *Alistipes* spp. has been shown to be associated with liver fibrosis, cardiovascular disease, cancer immunotherapy, cardiovascular disease, colitis, and depression [51]. The decrease of *Alistipes* spp. causes a reduction in short-chain fatty acids (SCFA), which further leads to a decrease in the levels of anti-inflammatory cytokines and decreased inhibition of Th17 cells, resulting in liver fibrosis and hepatocellular carcinoma [51]. Our results suggest that exposure to AFB_1_ may lead to an increase in *BF311* spp. and a decrease in *Alistipes* spp. populations, and the changes in these bacteria are related to liver dysfunction in sheep.

## 4. Conclusions

This study confirmed that oxidative stress, inflammatory injury, apoptosis, and gut microbiota are involved in the liver injury and liver dysfunction caused by AFB_1_ exposure in sheep. The study also offers a valuable reference for future research into the mechanism underlying the hepatotoxic effects of aflatoxin on sheep.

## 5. Materials and Methods

### 5.1. Toxin

AFB_1_ (>98% pure) was purchased from Pribolab Chemical Inc., Co. (Qingdao, China) and dissolved in 4% (*v*/*v*) ethanol.

### 5.2. Animals, Exposure Experiment

The study was mainly conducted at Henan Agricultural University’s Xuchang practical teaching base, China. Twelve Dorper RAMS with an average body weight of 22.34 ± 5.07 kg were individually identified by ear tag and randomly divided into two groups of three replicates and two sheep each. The sheep were immunized according to routine procedures. The control group received 4% ethanol (20 mL) through gavage, while the AFB_1_ group received AFB_1_ (1 mg/kg, dissolved in 20 mL 4% ethanol) (half of LD50) orally [8]. Following the gavage of AFB_1_, there was a 24 h fast from food and water, and surgical sampling was carried out 24 h later. The animal care and experimental procedures were approved by the Institutional Animal Welfare and Research Ethics Committee of Henan Agricultural University’s College of Veterinary Medicine (Zhengzhou, China) (Permit No: 17-0126, Year of approval: 2017). The experimental animals were kept under anesthesia during surgery and every effort was made to minimize their pain, suffering, and death.

### 5.3. The Color of Conjunctiva

A handheld colorimeter (#SR-62; Shenzhen 3nh Technology Co., Ltd., Shenzhen, China) was used to assess conjunctival color prior to before intragastric administration and surgical sampling. Reflectance spectrometry was used for the color determination using the CIELab method.

### 5.4. Sample Collection

Temperature, respiration, and heart rate were measured before and 24 h after intragastric administration. Rectal temperature and heart rate were measured using a mercury-in-glass thermometer (range 35–42 °C; accuracy ± 0.3) and stethoscope, respectively. By counting the movement of the abdominal muscles on both sides while breathing, the sheep’s breathing rate per minute was calculated. Blood samples were drawn from the jugular vein; serum was isolated through centrifugation at 4 °C for 15 min at 3000× *g* and stored at −20 °C. Fecal samples were collected from the sheep rectum before surgery and kept at −80 °C. The anterior abdominal midline of umbilicus was selected as the surgical incision. The liver was exposed by laparotomy, about 2 cm × 3 cm × 3 cm hepatic lobules were collected for liver evaluation, and clamp hemostasis or electrocautery hemostasis was used. The wound was strictly aseptic debridement; each layer of abdominal wall was closed and wrapped with elastic bandage. Part of the liver tissue was cut into 1 cm^3^ pieces and transferred in liquid nitrogen to the laboratory, where it was preserved at −80 °C for further analysis. The other portion was fixed for 24 h with 4% paraformaldehyde.

### 5.5. Serum Biochemistry

To determine the 16 biochemical indexes of the serum, a full-automatic biochemical analyzer (#SMT-120 V, Seamaty technology Co., Ltd., Chengdu, China) was used, and the reagent plate was obtained from Chengdu Polytech biological technology Co., Ltd., China. The following parameters were measured within 2 h: ALB, TP, CK, GLU, AMY, A/G, Ca, TBIL, AST, ALT, CRE, TG, P, BUN, and GLO.

### 5.6. Liver Histopathology

Paraformaldehyde-fixed liver tissue samples were washed and dehydrated in ethanol before being extracted with toluene and embedded in paraffin. For qualitative histological analysis, tissues were sectioned (5 μm) and stained with hematoxylin and eosin (H&E). Motic BA600-4 microscope was used to photograph the tissues (Motic, Xiamen, China).

### 5.7. Detection of Apoptosis

Paraffin slices of liver were dewaxed using xylene and anhydrous ethanol, then incubated in a 37 °C incubator for 22 min with protease K. TDT enzyme and dUTP were used to incubate in incubator at 37 °C for 2 h before dropping DAPI dye and incubating for 10 min at room temperature. A fluorescent microscope was used for photo imaging (Nikon Eclipse C1, Nikon, Japan). Apoptosis was indicated by green fluorescence.

### 5.8. Liver Function of Tissue

The liver tissue was homogenized to 10% by the dilution solution, and the activity of general marker enzymes like ALT, AST, and alkaline phosphatase (AKP) enzymes in liver tissue were assessed using kits (Nanjing Jiancheng Bioengineering Factory, Nanjing, China).

### 5.9. Liver Antioxidant Abilities

The activities of superoxide dismutase (SOD) and catalase (CAT), and the concentration of total antioxidant capacity (T-AOC) and malondialdehyde (MDA) in the liver tissue supernatant were detected by the kit (Nanjing Jiancheng Bioengineering Institute, Nanjing, China).

### 5.10. Extraction of Total RNA and Quantitative Real-Time PCR Analysis

The CFX96 real-time PCR detection system was used for real-time quantitative PCR (Bio-Rad, Munich, Germany). TRIZOL reagent (Takara Biotechnology Co., Ltd., Dalian, China) was used to extract total RNA from liver tissue, and Superscript II reverse transcriptase was used to prepare first strand cDNA (Roche, Basel, Switzerland). SYBR Green I PCR Master Mix (Vazyme Biotech Co.,Ltd, Nanjing, China) was used to determine mRNA levels, which were then calculated using the 2^−ΔΔCT^ method. Sangon Biotech (Shanghai, China) designed specific primers (Table S1) for inflammatory genes (*TNF-α*, *IL-1β*, *IL-6*, and *IL-10*), antioxidant genes (*SOD-1*, *SOD-2*), and apoptosis genes (*Bcl-2*, *Bax*, and *Caspase-3*) based on the NCBI database sequence (Table S1).

### 5.11. Extraction of Faecal DNA, PCR Amplification, and Illumina Sequencing

Following the manufacturer’s instructions, total fecal DNA was extracted using the Fast DNA SPIN extraction kits (MP Biomedicals, Santa Ana, CA, USA). To amplify the V3-V4 region of the 16S rRNA gene, forward primer 338F and reverse primer 806R were used. QIIME2 dada2 and R package (v3.2.0) were primarily used for sequence data analysis. The National Center for Biotechnology Information Sequence Read Archive now contains the sequence information for the 16S rRNA gene obtained in our study (PRJNA844551).

### 5.12. Statistical Analysis

To ensure accuracy and reproducibility, every experiment was performed at least three times. Graphpad Prism (version 7.0) was used to analyze all of the data, which was expressed as means ± standard deviation (SD). The significance of any differences between the experimental groups was assessed using Student’s *t*-test. Statistics were considered significant at *p* < 0.05.

## Data Availability

The National Center for Biotechnology Information Sequence Read Archive now contains the sequence information for the 16S rRNA gene obtained in our study (PRJNA844551).

## Supplementary Materials

The following supporting information can be downloaded at: https://www.mdpi.com/article/10.3390/toxins14120840/s1, Table S1: Primer sequences for qPCR analysis; Table S2: Effects of AFB_1_ exposure on body temperature, breathing, and heart rate of sheep; Table S3: Effects of AFB_1_ exposure on serum biochemistry of sheep. Figure S1: Effects of AFB_1_ exposure on conjunctival color.
