# Supplementary material for: Aflatoxin B1 Exposure in Sheep: Insights into Hepatotoxicity Based on Oxidative Stress, Inflammatory Injury, Apoptosis, and Gut Microbiota Analysis
Source: Toxins (Basel). 2022 Dec 1;14(12):840. doi: 10.3390/toxins14120840 (PMC9787800; doi:10.3390/toxins14120840)
Supplement: Supplementary file 1 [file toxins-14-00840-s001.zip › toxins-2046497-supplementary.pdf]

toxins

Supplementary Materials: Aflatoxin B_1 Exposure in Sheep: Insights into Hepatotoxicity Based on Oxidative Stress, Inflammatory Injury, Apoptosis, and Gut Microbiota Analysis

Yuzhen Sui, Ying Lu, Shoujun Zuo, Haidong Wang, Xiaokun Bian, Guizhen Chen, Shucheng Huang, Hongyu Dai, Fang Liu and Haiju Dong

Table S1. Primer sequences for qPCR analysis.

Gene	Gene ID	Sequence（5′-3′）	Fragment length (bp)
β-actin	NM_001009784.3	F:GCTCTCTTCCAGCCTTCCTT	101
		R:AGGTCTTTGCGGATGTCG	
TNF-α	XM_012100437.3	F:CCCAACTCCCTCTGTTTATGTT	102
		R:GGACACCTTGACCTCCTGAATA	
IL-1β	NM_001009465.2	F:TCTCCCTAAAGAAGCCATAC	151
		R:AGCGTCTCAGCACGAATA	
IL-6	NM_001009392.1	F:ACGAGTGGGTAAAGAACGCAAAGG	99
		R:GCCGCAGCTACTTCATCCGAATAG	
IL-10	NM_001009327.1	F:GGTGATGCCACAGGCTGAGAAC	143
		R:GCTCCACCGCCTTGCTCTTG	
SOD-1	NM_001145185.2	F:TTCTGCGGCGTTTCCTTGTCTG	138
		R:CGAAGCGGATGGTGCCTTGC	
SOD-2	NM_001280703.1	F:GGCAGCGTCCACCATGTTGTC	144
		R:GCGTTGATGTGCGGCTCCAG	
Caspase-3	XM_027962551.1	F:CCCACTGCAGCAACATTAATCC	150
		R:CCGGAGTCCACTGATTTGCT	
Bax	XM_027978594.1	F:CGCATTGGAGATGAATTGGACAGTAAC	128
		R:CAGTTGAAGTTGCCGTCGGAAAAC	
Bcl-2	XM_027960877.1	F:TGTGGATGACCGAGTACCTGAACC	127
		R:AGAGACAGCCAGGAGAAATCAAACAG	

Note: F, Forward primer; R, Reverse primer. β-actin, reference gene.

Table S2. Effects of AFB1 exposure on body temperature, breathing, and heart rate of sheep.

Factors	Item	Pro-test	Post-test	D-value
Body temperature	Control group	39.25±0.39[a]	39.20±0.55[a]	-0.05±0.29
	AFB1 group	38.88±0.50[a]	39.53±0.78[a]	0.65±0.23
	P-value	0.280	0.519	0.043
Breathing	Control group	17.75±2.99[a]	18.25±2.98[a]	0.5±2.52
	AFB1 group	13.25±4.65[a]	12.75±4.03[a]	-0.5±1.0
	P-value	0.280	0.071	0.488
Heart rate	Control group	56.75±5.56[a]	73.50±12.79[a]	16.75±13.25
	AFB1 group	67.25±11.30[a]	57.50±8.70[a]	-9.75±11.45
	P-value	0.146	0.084	0.022

Note: Mean values with same superscripts in a raw have no significant (P> 0.05), acompared pro-test to post-test (n = 6/group); D-value: The differences between post-intervention and pre-intervention.

Table S3. Effects of AFB1 exposure on serum biochemistry of sheep.

Item	Control group	AFB1 group	*P*-value
TP (g/L)	69.03±5.87	63.17±9.10	0.214
ALB (g/L)	24.72±2.88	21.18±3.66	0.093
GLO (g/L)	44.30±4.53	41.97±7.07	0.512
A/G	0.56±0.08	0.51±0.01	0.397
TBIL (umol/L)	1.6±0.3[a]	2.85±0.47[b]	0.002
AST (U/L)	126.83±22.38	152±37.67	0.190
ALT (U/L)	14.83±10.80	20.33±6.28	0.582
AMY (U/L)	15.83±10.21	12.75±10.90	0.660
TG (mmol/L)	0.44±0.15	1.32±1.47	0.470
CRE (umol/L)	57.20±7.36	63.20±11.45	0.306
BUN (mmol/L)	8.37±2.78	9.57±4.64	0.599
BUN/CRE	149.97±55.98	152.77±65.03	0.938
Ca (mmol/L)	2.10±0.063[a]	1.96±0.083[b]	0.006
P (mmol/L)	2.56±0.31	2.14±0.52	0.119
GLU (mmol/L)	4.67±3.18	3.30±2.05	0.513
CK (U/L)	262±262.46	512.83±648.79	0.411

Note: [a,b]Mean values with different superscripts in a raw differ significantly ($P < 0.05$, n = 6/group).

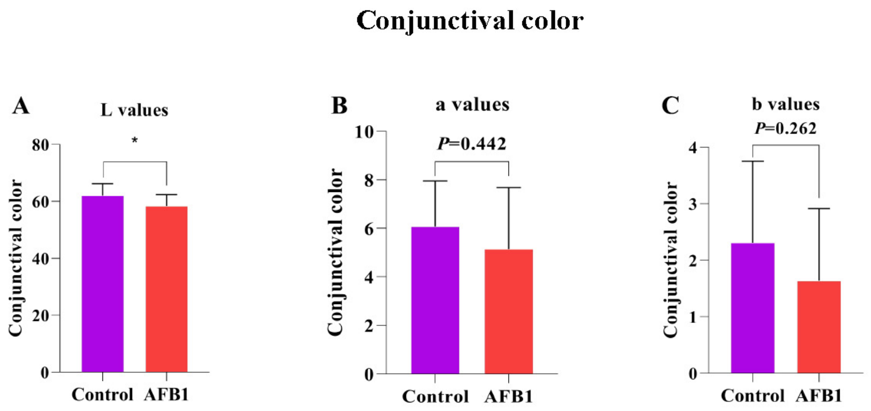

Figure S1. Effects of AFB1 exposure on conjunctival color. (A–C) Conjunctival color [lightness (L values), redness (a values), yellowness (b values)]. Data represent means±s.d., Student's t-test. Statistics were considered significant at $P < 0.05$.* $P < 0.05$.
